# Seasonal variation of phytoplankton community assembly processes in Tibetan Plateau floodplain

**DOI:** 10.3389/fmicb.2023.1122838

**Published:** 2023-02-20

**Authors:** Zhenyu Huang, Baozhu Pan, Janne Soininen, Xinyuan Liu, Yiming Hou, Xing Liu

**Affiliations:** ^1^State Key Laboratory of Eco-Hydraulic in Northwest Arid Region of China, Xi’an University of Technology, Xi’an, Shanxi, China; ^2^Department of Geosciences and Geography, University of Helsinki, Helsinki, Finland

**Keywords:** phytoplankton community, community assembly, oxbow lakes, Tibetan Plateau, flood pulse, hydrological connectivity

## Abstract

Uncovering the mechanisms underlying phytoplankton community assembly remains a major challenge in freshwater ecology. The roles of environmental filtering and spatial processes in shaping phytoplankton metacommunity in Tibetan floodplain ecosystems under various hydrological conditions are still unclear. Here, multivariate statistics and a null model approach were used to compare the spatiotemporal patterns and assembly processes of phytoplankton communities in the river-oxbow lake system of Tibetan Plateau floodplain between non-flood and flood periods. The results showed that phytoplankton communities had significant seasonal and habitat variations, with the seasonal variations being more remarkable. Phytoplankton density, biomass, and alpha diversity were distinctly lower in the flood than non-flood period. The habitat differences (rivers vs. oxbow lakes) in phytoplankton community were less pronounced during the flood than non-flood period, most likely due to the increased hydrological connectivity. There was a significant distance–decay relationship only in lotic phytoplankton communities, and such relationship was stronger in the non-flood than flood period. Variation partitioning and PER-SIMPER analysis showed that the relative role of environmental filtering and spatial processes affecting phytoplankton assemblages varied across hydrological periods, with environmental filtering dominating in the non-flood period and spatial processes in the flood period. These results suggest that the flow regime plays a key role in balancing environmental and spatial factors in shaping phytoplankton communities. This study contributes to a deeper understanding of ecological phenomena in highland floodplains and provides a theoretical basis for floodplain ecosystem maintenance and ecological health management.

## 1. Introduction

Phytoplankton, as major primary producers, play a vital role in maintaining aquatic ecosystem stability and functioning (Padisák et al., 2006). The mechanisms of phytoplankton metacommunity assembly and maintenance have always been a key issue in aquatic ecology, particularly in lotic and lentic ecosystems (Huszar et al., 2015; Guelzow et al., 2016; Chaparro et al., 2018). Niche-based (e.g., environmental filtering and biotic interactions) and neutral processes (e.g., spatial processes; namely, mass effects and dispersal limitation) are considered the most important complementary mechanisms driving aquatic community assembly (Heino, 2013; Lima-Mendez et al., 2015; Liu et al., 2015). Niche-based processes emphasize that metacommunities are shaped by deterministic abiotic (e.g., nutrients, light, and temperature) and biotic (e.g., species competition, grazing, and predation) factors that depend upon differences within and among habitat environments and the fitness of their species (Leibold et al., 2004). Concerning neutral processes, dispersal limitation and ecological drift including random births and deaths are considered the dominant stochastic factors responsible for a metacommunity’s structure (Beisner et al., 2006; Naselli-Flores and Padisák, 2016).

There are number of ways to quantitatively investigate how environmental filtering and spatial processes impact aquatic metacommunities (Gallego et al., 2014; Heino et al., 2015; Santos et al., 2016). The key to studying stochastic processes in community assembly and biodiversity lies in the measurement of species dispersal, but this is difficult to accomplish in the field simultaneously for multiple taxa. Accordingly, their spatial structure may be used as an effective proxy for stochastic effects such as dispersal (Beisner et al., 2006; Lindström and Langenheder, 2012; Heino and Tolonen, 2017). For example, phytoplankton community assembly mechanisms have been studied in marine, river, and lake environments recently. Studies suggest that environmental filtering, rather than spatial processes, predominantly drives the structure of phytoplankton communities (Cottenie, 2005; Zhao et al., 2021). However, such investigations remain limited in river-lake networks in highland floodplains even if they represent one of the most interesting systems to study spatial ecology of algae.

River-lake systems harbor complex biogeochemical variability, with their chemical and hydrological characteristics influenced by seasonality coupled to other factors, such as agriculture, industry, and dam construction (Marshall et al., 2006; Lansac-Tôha et al., 2016). In addition, the spatial structure in terms of topography, geomorphology, and river network morphology will jointly impact community assemblage dynamics in river-lake systems. Normally, dispersal limitation is stronger in lakes than rivers, since lakes are typically located at the edge of the river network and constitute the most isolated areas in the river landscape (Brown and Swan, 2010; Tonkin et al., 2018). By contrast, the mainstream is usually close to the center of a river network and has a high degree of water connectivity, which facilitates the migration, dispersal, and gene exchange of species between habitats, generating a stronger mass effect in rivers than in lakes (Besemer et al., 2013). Furthermore, the potential influencing factors driving metacommunity assembly can vary across hydrological periods (Chaparro et al., 2018; Isabwe et al., 2018). For example, the hydrological connectivity between river and lake habitats in floodplains during the dry season can lead to high community and environmental variability. Environmental filtering may be the dominant factor in community dynamics in the low flow season. During high-flow seasons, flood pulses increase connectivity between habitats, leading to homogenization of communities or environmental conditions, where spatial processes (mass effects) may constructing community dynamics (Li et al., 2022). Currently, there is still lacking of empirical evidence on the mechanisms shaping phytoplankton community in highland floodplains during their non-flood and flood periods.

The White River is one of the largest tributaries in the Yellow River source region on the Tibetan Plateau floodplain in China, where it plays an essential role in the ecological replenishment, security, and stability of the Yellow River (Zhou et al., 2019; Wang et al., 2020). The White River consists of many small tributaries, with typical geomorphic units, namely, oxbow lakes formed due to neck or chute cutoffs *via* river bank erosion and overflow floods (Leopold and Wolman, 1957). These oxbow lakes, generally U- or Ω- shaped, belong to the abandoned channel and are thus isolated from the main channel (Delhomme et al., 2013). Oxbow lakes are usually divided into three types based on their hydrological connectivity to the main river channel. Lotic lakes are permanently connected to the active channel at both ends, whereas semi-lotic lakes are connected at only the downstream end, and lentic lakes are temporarily connected during high flows (Kobus et al., 2016). Collectively, these differing oxbow lakes provide diverse habitats supporting floodplain biodiversity, in addition to other social-economic functions such as fisheries, recreation, and flood control (Acreman et al., 2007).

Irrespective of their type, the hydrological connectivity of oxbow lakes to the river is greatly increased by the flood pulse that occurs during high flow periods (Wang et al., 2020). Some studies have demonstrated the importance of hydrological control for the distribution of plankton communities in other river-oxbow lake systems worldwide (Nabout et al., 2006; Townsend, 2006; Butler et al., 2007; Mihaljević et al., 2009). Isabwe et al. (2018) reported that environmental factors play a more important role than spatial factors in phytoplankton assembly in both dry and wet seasons, although the importance of spatial factors increased during high water level. Devercelli et al. (2016) found that stochastic process to dominate phytoplankton structure in Paraná River floodplain. Yet the phytoplankton community assembly mechanisms of the Tibetan floodplain ecosystem in relation to seasonal hydrological variations are still poorly understood. Here, we explored phytoplankton community structure and assembly processes, along with environmental and spatial gradients, in the White River and its oxbow lakes during non-flood and flood periods. We had two primary questions: (1) Do phytoplankton communities in river-oxbow lake system show distinct spatiotemporal patterns across hydrological periods? (2) To what extent do environmental filtering and spatial processes explain phytoplankton community assembly in the highland river-oxbow lake system during contrasting hydrological periods?

## 2. Materials and methods

### 2.1. Study area

**FIGURE 1:**
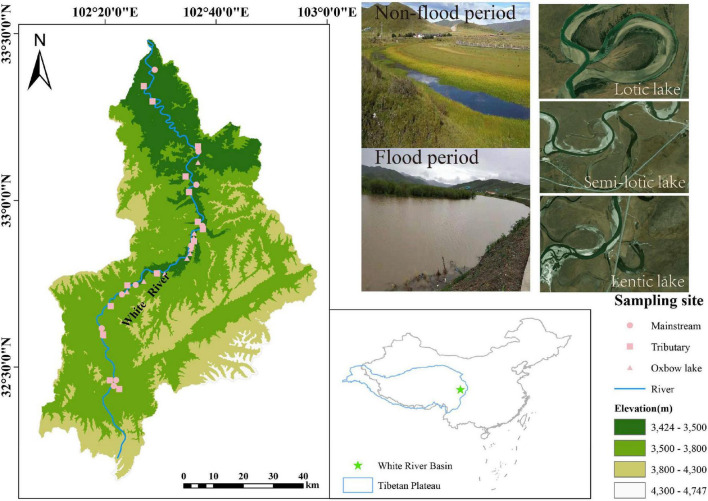
Spatial distribution of sampling points in the White River Basin during the non-flood and flood periods.

The White River Basin (33°00′–33°30′N, 102°00′–103°00′E) is located within the Zoige wetland on the northeastern Tibetan Plateau of China, with a mean elevation >3,500 m a.s.l (Figure 1). The study area lies within the continental cold temperate climate zone, having a mean annual air temperature of 0.7–1.1°C and a mean annual precipitation of 600–700 mm. Approximately 90% of precipitation is concentrated in the flood period (May to late August) (Wang et al., 2020). The hydrological conditions here differ markedly between non-flood and flood periods: mean monthly runoff ranged from 75.79 m^3^/s in September 2019 to 166.96 m^3^/s in June 2020 (based on data from the Tangke Hydrological Station located at the downstream of the White River Supplementary Figure 1).

The White River (270 km) contributes 10–20% of the annual runoff entering the Yellow River (Xiang et al., 2009). As the White River meanders through the Zoige wetland, oxbow lakes commonly form due to frequent meander cutoffs caused by wide valleys in the wetland (Wang et al., 2020). Because of its harsh alpine climate, population density in White River Basin is considerably low, at 5.8 persons/km^2^. Consequently, the aquatic ecosystem is barely impacted by human activities, leaving natural evolution trends intact (Wang et al., 2020). Given its unique environmental and biological conditions, the White River Basin provides a perfect opportunity for examining the spatiotemporal patterns and drivers of phytoplankton metacommunity assembly in a highland river-oxbow lake system.

### 2.2. Sampling and measurement of environmental variables

Field sampling was carried out in September 2019 (non-flood period) and June 2020 (flood period). In either season, the sampling duration lasted no more than 15 days. Thirty-six sampling points were selected across the White River Basin: 10 in its mainstream, 14 in its tributaries, and 12 in its oxbow lakes (Figure 1). Six subsurface water samples (three for chemical analysis and three for phytoplankton analysis) were collected using 1 L polyethylene bottles at each sampling point, for 432 samples in total.

Environmental variables (seven physical and four chemical parameters) were measured in the field and laboratory. *In situ* measurements of water temperature (WT), electrical conductivity (Cond), dissolved oxygen (DO), and pH level were taken with an YSI multi-parameter analyzer (YSI Corp., Yellow Spings, OH, USA). Water depth (WD) was measured using a standard tape and flow velocity (V) recorded by a Global Water FP211 direct reading flow meter (Global Water Instrumentation, Sunnyvale, CA, USA). Turbidity (Tur) was measured using a Hach 2100Q portable turbidity meter (Hach, Loveland, CO, USA). Water samples for chemical analysis were stored in ice bags and kept in darkness until moved to the laboratory for chemical analysis. Total phosphorus (TP), total nitrogen (TN), ammonia nitrogen (NH_4_-N), and nitrate nitrogen (NO_3_-N) concentrations were determined based on the standard methods for surface water environmental quality of China (Wei et al., 1989; Huang et al., 1999).

### 2.3. Phytoplankton identification and biomass analysis

Three water samples were collected (using 1 L polyethylene bottles) at each sampling site and fixed *in situ* with 15 mL of 5% Lugol’s iodine solution. After settlement for 48 h, the supernatant of preserved phytoplankton samples was slowly siphoned with a thin rubber tube, leaving 30 mL of settled phytoplankton and were transferred into 50 mL sample bottles. The polyethylene bottles were rinsed two or three times with the supernatant, and the phytoplankton sample volume was then brought to about 50 ml in the sample bottles. Finally, 1–2 mL of 40% formaldehyde was added to samples for long-term preservation (Ding et al., 2022). A 0.1-mL subsample was transferred to the Sedgewick Rafter counting chamber and phytoplankton density was examined under an inverted microscope (Zeiss, Jena, Germany). We observed 100 random fields at 400× magnification, and each sample was examined three times. For diatoms, permanent slides were prepared following Wu et al. (2021) and inspected with an optical microscope at 1,000× magnification. The identification and quantification of phytoplankton species were according to the methodology described by Hu and Wei (2006) based on the morphological structure and habitat type of phytoplankton. Phytoplankton was identified to the lowest taxonomic level possible. The phytoplankton biomass of each sample was estimated using phytoplankton biovolume. We randomly selected 30–50 individuals of each specie and measured their length, height and diameter according to the most approximate geometric shape to calculate the average biovolume (Hillebrand et al., 1999). The average biovolume and abundance-based results of each species were used to estimate biomass using the conversion factor (1 μm^3^ = 1 pg) (Wetzel and Likens, 2000).

### 2.4. Data analysis

Species richness and the Shannon–Wiener index were used to analyze alpha diversity of phytoplankton communities. One-way repeated-measures analysis of variance (ANOVA) was implemented in the SPSS v25.0 statistic software (IBM, Armonk, NY, USA) to reveal the possible differences in the environmental variables and phytoplankton density, biomass, and alpha diversity between the two hydrological periods and among the three habitat types. Before ANOVAs, we checked the normality of environmental and phytoplankton data and transformed non-normal variables. The permutation multivariate dispersion analysis (PERMDISP) was applied to analyze environmental heterogeneity of the study area between seasons. With respect to beta diversity, the analysis of similarities (ANOSIM) was applied to evaluate the similarities of phytoplankton communities among different habitats in each period. Then the community similarities were visualized by non-metric multidimensional scaling (NMDS) based on Bray–Curtis dis-similarity matrix. A Venn diagram was used to quantitatively analyze and depict the co-occurrence of phytoplankton species between rivers and oxbow lakes (Lin et al., 2016).

Correlation analysis was conducted using the Euclidean distance (km; straight-line distance between sampling points in two-dimensional space) to determine the distance–decay relationship (DDR) in phytoplankton communities based on Bray–Curtis similarity matrix (calculated as 1–Bray–Curtis dis-similarity) (Jiang et al., 2017). We used phytoplankton abundance data to calculate the matrix. We considered the Euclidean distance as the major component of phytoplankton dispersal that occurred *via* aerial and water dispersal. Principal coordinates of neighbor matrices (PCNM) were performed to create spatial factors using the “vegan” package in the R platform v4.0.3 (R Developement Core Team, 2015). Then, distanced-based redundancy analysis (dbRDA) were applied to select the key environmental and spatial variables relating to phytoplankton community. Before analyses, phytoplankton abundance data were Hellinger-transformed. To eliminate collinearity, the environmental variables with a variance inflation factor (VIF) > 20 were removed. The significant environmental and spatial factors were determined based on the forward selection procedure. Variation partitioning analysis (VPA) was applied to quantitatively describe the relative contribution of environmental filtering and spatial processes for phytoplankton community assembly. VPA analysis is widely used to identify the driving processes of metacommunity structure in freshwater studies (e.g., Heino et al., 2014; Niño-García et al., 2016; Isabwe et al., 2018). The total variation in phytoplankton structure was divided into pure environmental (E), pure spatial (S), shred (E∩S), and unexplained (U) fractions. VPA was carried out by using the *varpart* function from the R package “vegan.” In addition, a permutation-based algorithm (PER-SIMPER) (Gibert and Escarguel, 2019) was applied to further explore the first-order processes (i.e., niche- and/or dispersal-based processes) underlying a given set of phytoplankton communities.

## 3. Results

### 3.1. Water environmental variables

Most of the water environmental variables tested differed significantly between hydrological periods (*P* < 0.05; Table 1). The mean concentrations of TN, TP, and NH_4_^+^N in the flood period (0.45, 0.04, and 0.24 mg/L, respectively) were ∼50% lower than those in the non-flood period (0.94, 0.07, and 0.73 mg/L, respectively; Supplementary Table 1). Conversely, several physical environmental variables, including WD, Tur, and WT, were higher in the flood period; the mean Tur values increased sharply, from 110 NTU in the non-flood period to 347 NTU in the flood period. During both hydrological periods, the mean values for V, WT, Cond, DO, and Tur showed significant differences among the three habitats. Those of V, DO, and Tur were distinctly higher in the mainstream and tributaries than in oxbow lakes. Furthermore, the interaction effects between period and habitat were significant for the TN and NH_4_^+^-N concentrations. The results of PERMDISP analysis showed that the environmental heterogeneity was stronger in the non-flood period than in the flood period (Supplementary Figure 2).

**TABLE 1:** Repeated-measured ANOVA results of water environmental variables in three different habitats of the White River Basin across two hydrological periods.

Variable	*F*	*P*-value	Ranking (*post-hoc* tests or contrast)
**Water depth (cm)**			
Between-subjects (habitat)	2.09	0.139	
Period	14.46	**0**.**001**	Flood period > non-flood period
Period × habitat	0.95	0.397	
**Flow velocity (m/s)**			
Between-subjects (habitat)	30.85	**<0**.**001**	Tributaries, mainstream > oxbow lakes
Period	0	0.993	
Period × habitat	1.47	0.245	
**Water temperature (°C)**			
Between-subjects (habitat)	3.40	**0**.**046**	Oxbow lakes > mainstream, tributaries
Period	1.39	0.247	
Period × habitat	0.39	0.680	
**Conductivity (μ S/cm)**			
Between-subjects (habitat)	3.75	**0**.**034**	Tributaries > oxbow lakes, mainstream
Period	15.74	**<0**.**001**	flood period > non-flood period
Period × habitat	0.63	0.540	
**Dissolved oxygen (mg/L)**			
Between-subjects (habitat)	6.40	**0**.**005**	Mainstream, tributaries > oxbow lakes
Period	27.33	**<0**.**001**	Non-flood period > flood period
Period × habitat	0.11	0.90	
**pH**			
Between-subjects (habitat)	0.52	0.598	
Period	11.47	**0**.**002**	Non-flood period > flood period
Period × habitat	0.61	0.550	
**Turbidity (NTU)**			
Between-subjects (habitat)	6.39	**0**.**005**	Mainstream > tributaries > oxbow lakes
Period	27.36	**<0**.**001**	Flood period > non-flood period
Period × habitat	0.11	0.898	
**Total N (mg/L)**			
Between-subjects (habitat)	0.05	0.954	
Period	164.84	**<0**.**001**	Non-flood period > flood period
Period × habitat	12.16	**<0**.**001**	
**NH_4_^+^-N (mg/L)**			
Between-subjects (habitat)	1.20	0.316	
Period	148.20	**<0**.**001**	Non-flood period > flood period
Period × habitat	5.04	**0**.**012**	
**NO_3_^–^-N (mg/L)**			
Between-subjects (habitat)	2.43	0.104	
Period	1.49	0.231	
Period × habitat	0.06	0.938	
**Total *P* (mg/L)**			
Between-subjects (habitat)	2.03	0.147	
Period	39.96	**<0**.**001**	Non-flood period > flood period
Period × habitat	1.67	0.204	

*P*-values < 0.05 are in bold.

### 3.2. Phytoplankton composition, density, and biomass

Overall, 172 phytoplankton taxa belonging to seven phyla and 79 genera were identified in the study area. These consisted of 94 taxa of Bacillariophyta (54.6%), 44 of Chlorophyta (25.6%), 14 of Cyanophyta (8.1%), 13 of Euglenophyta (7.6%), 4 of Dinophyta (2.3%), 2 of Cryptophtya (1.2%), and 1 of Chrysophyta (0.6%; Supplementary Figure 3). Additionally, the highest richness values were found in the oxbow lakes in both periods, accounting for about 90% of the total taxa numbers in the river-oxbow lake system.

**FIGURE 2:**
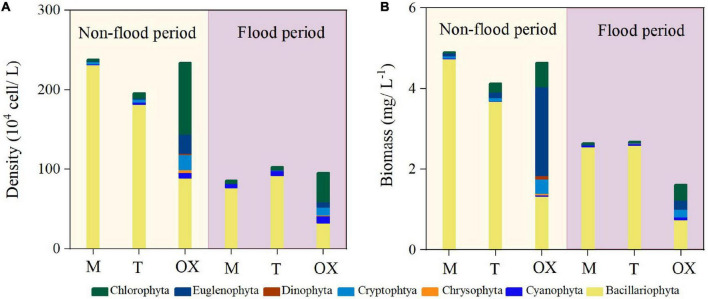
Phytoplankton mean **(A)** density and **(B)** biomass in the mainstream (M), tributaries (T), and oxbow lakes (OX) during two contrasting hydrological periods of the White River Basin.

In the non-flood period, total phytoplankton density ranged from 195.71 × 10^4^ to 237.96 × 10^4^ cells L^–1^, and the total biomass ranged from 4.13 to 4.65 mg L^–1^ among the three habitats (Figure 2). In the flood period, the phytoplankton density ranged from 85.63 × 10^4^ to 103.3 × 10^4^ cells L^–1^, and the biomass varied between 1.61 and 2.69 mg L^–1^ among the three habitats. The mean cell density and biomass of phytoplankton were significantly higher (*P* < 0.01) in the non-flood than flood period (Table 2). Additionally, Bacillariophyta was the most dominant phylum present in the mainstream and tributary sites during either hydrological period, accounting for ∼90% of the total cell density (Supplementary Figure 3). Phytoplankton taxa composition was more uniform in the oxbow lakes, where Chlorophyta accounted for the highest mean proportion (38.3%) of total cell density in both periods, followed by Bacillariophyta (35.9%), Cryptophyta (10.4%), and Cyanophyta (9.8%).

**TABLE 2:** Repeated-measured ANOVA results of phytoplankton density, biomass, and alpha diversity in different habitats of the White River Basin across two hydrological periods.

Variable	*F*	*P*-value	Ranking (*post-hoc* tests or contrast)
**Density (10^4^ cells L^–1^)**			
Between-subjects (habitat)	0.36	0.702	
Period	7.94	**0**.**008**	Non-flood period > flood period
Period × habitat	0.08	0.927	
**Biomass (mg L^–1^)**			
Between-subjects (habitat)	0.31	0.735	
Period	15.48	**<0**.**001**	Non-flood period > flood period
Period × habitat	1.05	0.060	
**Species richness**			
Between-subjects (habitat)	6.15	**0**.**005**	Mainstream, tributaries > oxbow lakes
Period	8.67	**0**.**006**	Non-flood period > flood period
Period × habitat	16.33	**<0**.**001**	
**Shannon–Wiener diversity**			
Between-subjects (habitat)	6.21	**0**.**005**	Tributaries, mainstream > oxbow lakes
Period	0.13	0.725	
Period × habitat	0.01	0.993	

*P*-values < 0.05 are in bold.

### 3.3. Alpha and beta diversity of phytoplankton

**FIGURE 3:**
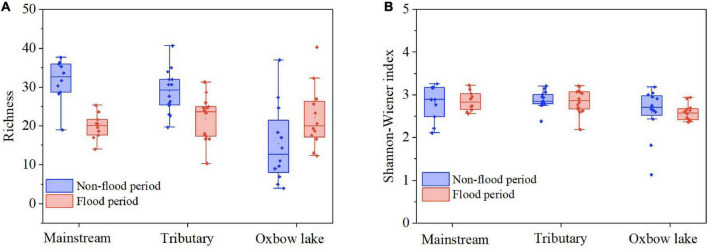
Alpha diversity of phytoplankton communities in terms of panel **(A)** species richness and **(B)** Shannon–Wiener index in different habitats of the White River Basin during two contrasting hydrological periods.

Mean species richness of phytoplankton in each of the three habitats was significantly higher (*P* < 0.01) in the non-flood than flood period (Figure 3 and Table 2). Yet it is worth noting that species richness of oxbow lakes was greater in the flood than non-flood period. The mean richness was highest in the mainstream and lowest in oxbow lakes; the mean Shannon–Wiener index was highest in tributaries and lowest in oxbow lakes. In addition, it was found that seven genera—*Phacus*, *Gyrosigma*, *Spirulina*, *Anabaenopsis*, *Nostoc*, *Strombomonas*, *Pleodorina*—shared between the mainstream and oxbow lakes during the flood period only, all of which were absent in the mainstream during the non-flood period. Based on the genera uniquely shared between the mainstream and oxbow lakes, allochthonous genera (e.g., *Phacus* and *Gyrosigma*) from the oxbow lakes potentially accounted for 2.8 and 16.3% of mainstream’s total phytoplankton diversity during the non-flood and flood periods, respectively (Supplementary Figure 4).

**FIGURE 4:**
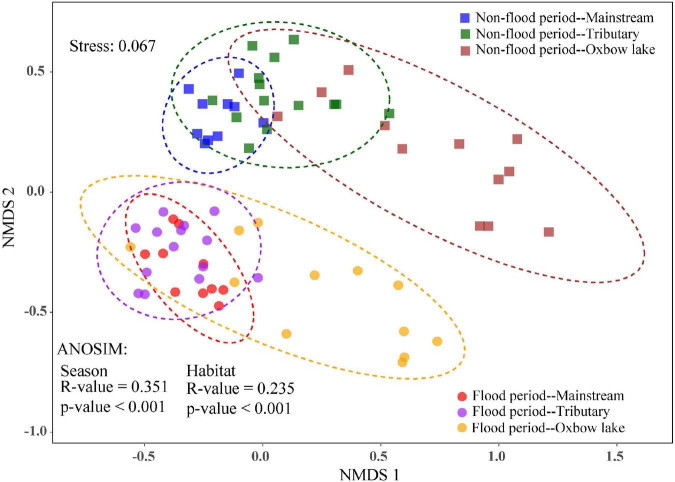
Beta diversity of phytoplankton communities among different habitats in non-flood and flood periods of the White River Basin based on non-metric multidimensional scaling (NMDS) and analysis of similarities (ANOSIM).

The NMDS analysis showed shifts in phytoplankton community composition among seasons and habitats (Figure 4), whereas the effects of season (ANOSIM: *R* = 0.351, *P* < 0.001) was stronger than that of habitat (ANOSIM: *R* = 0.235, *P* < 0.001). During the non-flood period, the beta diversity of phytoplankton showed significant difference between the river and the oxbow lakes, with the sample dispersion ellipses well separated between the two groups. By contrast, in the flood period, the sample dispersion ellipses partly overlapped between the river (mainstream and tributaries) and oxbow lake groups. The consistency of the results for beta diversity was confirmed by ANOSIM analysis (Supplementary Table 2). The community-level differences of phytoplankton among the three habitats in the flood period were significant (*R* = 0.338, *P* < 0.01), but relatively smaller than in the non-flood period (*R* = 0.389, *P* < 0.01). Furthermore, community-level differences between rivers and oxbow lake sites were larger in the non-flood period (*M* vs. OX: *R* = 0.434, *P* < 0.01; Tr vs. OX: *R* = 0.423, *P* < 0.01) than in the flood period (*M* vs. OX: *R* = 0.350, *P* < 0.01; Tr vs. OX: *R* = 0.389, *P* < 0.01; Supplementary Table 2).

### 3.4. Spatial heterogeneity of phytoplankton communities

**FIGURE 5:**
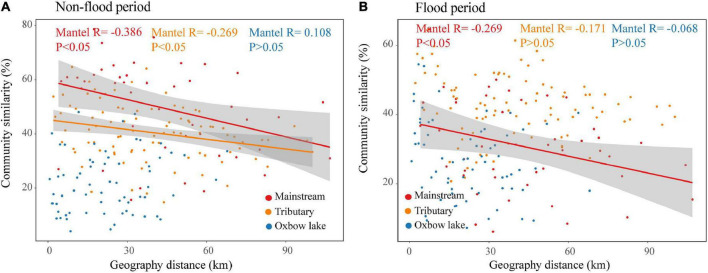
Distance–decay relationships between phytoplankton community similarities and geographic distance in the river-oxbow lake system of the White River Basin during the **(A)** non-flood and **(B)** flood periods.

The DDRs for phytoplankton community similarity vs. geographic distance in the river groups (mainstream and tributaries) were negative and significant (*P* < 0.05) in the non-flood period (Figure 5). Moreover, a stronger DDR was found in the mainstream than in the tributaries. The community similarity significantly decreased with geographic distance only in mainstream during the flood period. However, irrespective of the period, community similarity as a function of geographic distance did not yield a significant DDR in oxbow lakes. The results of Mantel tests showed that in non-flood season, both geographical and environmental distance had significant effects on river (mainstream and tributaries) communities, while only environmental distance had significant effects on oxbow lake communities (Supplementary Table 3). In the flood period, only geographical distance had significant influence on mainstream communities.

### 3.5. Relative role of environmental and spatial factors on phytoplankton community assembly

**FIGURE 6:**
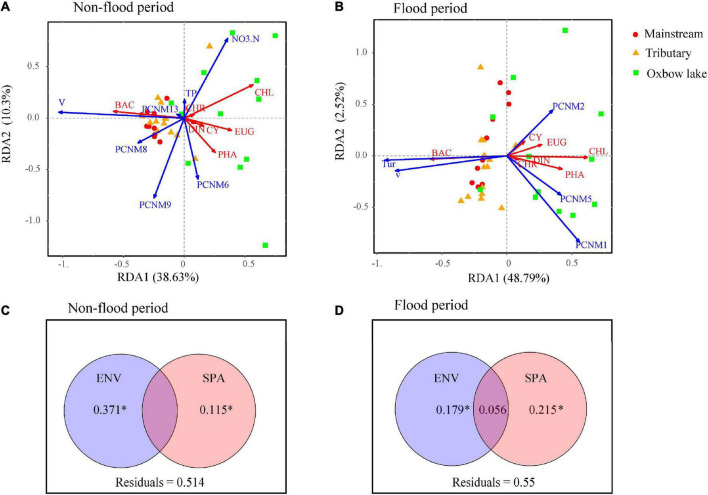
The relative contribution of local environmental (ENV) and spatial (SPA) variables to the variation of phytoplankton community dynamics (at phyla level) in the river-oxbow lake system of the White River Basin during the **(A,C)** non-flood and **(B,D)** flood periods. CYA, Cyanophyta; CHL, Chlorophyta; BAC, Bacillariophyta; EUG, Euglenophyta; CRY, Cryptophyta; CHR, Chrysophyta; EUG, Euglenophyta. Adjusted *R*^2^ values are shown. *P < 0.05 based on 999 permutations.

The dbRDA results revealed that during the non-flood period, the phytoplankton community dominated by Bacillariophyta in the mainstream and tributaries was mainly influenced by one environmental variable (V) and three spatial factors (PCNM 8, PCNM 9, and PCNM 13; Figure 6). In the oxbow lakes, the phytoplankton community dominated by non-Bacillariophyta phyla (i.e., Euglenophyta and Chlorophyta) was primarily influenced by two environmental variables (TP and NO_3_^–^-N) and one spatial factor (PCNM 6). In the flood period, the phytoplankton community in river sites was primarily influenced by local environmental variables (V and Tur), while the phytoplankton in oxbow lakes had strong relationships with purely spatial factors (PCNM 1, PCNM 2, and PCNM 5).

**FIGURE 7:**
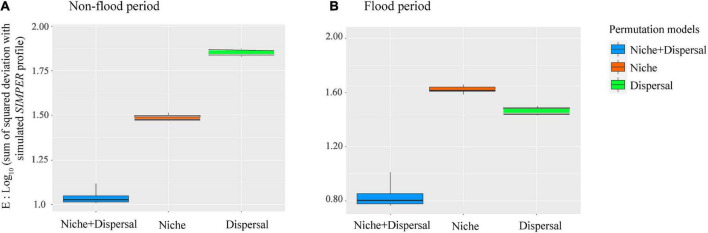
PER-SIMPER analysis revealing the relevance of niche-based and stochastic processes for phytoplankton community assembly “during the **(A)** non-flood and **(B)** flood periods” in the study area. The lower the *E* value, the closer the simulated profile to the empirical simper model.

The VPA results indicated that compared with spatial factors, environmental variables explained more variance of phytoplankton communities in the non-flood period (Figure 6C). The variation explained by pure environmental variables in the non-flood period (37.1%) surpassed that in the flood period (17.9%), while the explanatory power of pure spatial factors was increased from 11.5% in the non-flood period to 21.5% in the flood period. In addition, interactions between environmental and spatial factors increased considerably, going from 0 in the non-flood period to 5.6% in the flood period. The PER-SIMPER analysis results also indicated that niche- and dispersal-based processes jointly shaped phytoplankton communities in both periods (Figure 7). Crucially, the explanatory power of niche-based processes (environmental processes) was greater than that of stochastic processes (spatial processes) in the non-flood period. However, the influence of spatial processes surpassed environmental processes in the flood period.

## 4. Discussion

Previous studies on metacommunity dynamics mainly concentrated in snapshot surveys of a single hydrological period, while overlooked the impact of seasonal and hydrological variability for metacommunities, especially for those inhabiting in floodplain systems with high environmental gradients (Chaparro et al., 2018; Isabwe et al., 2018). The present study investigated the spatiotemporal distribution patterns and inferred potential assembly mechanism of phytoplankton communities in the Tibetan Plateau floodplain. This study expected to contribute to a more favorable extrapolation of ecological phenomena and to provide corresponding management and conservation recommendations for Tibetan floodplain ecosystem.

### 4.1. Assembly processes of phytoplankton communities in river-oxbow lake system of highland floodplain

Recent studies have shown that environmental filtering drives plankton community assembly at different seasons in rivers, estuaries and lakes that are highly disturbed by human activities (Isabwe et al., 2018; Zhou et al., 2021; Shen et al., 2022). However, our results indicated that phytoplankton assembly processes showed obvious seasonal change in the near-natural highland floodplain. Environmental filtering and spatial processes dominated phytoplankton assembly in the non-flood and flood periods, respectively. This finding was further confirmed by the PER-SIMPER analysis. In the study area, the environmental heterogeneity in the non-flood period was higher than that in flood season. Environmental factors such as flow velocity and nutrient concentrations feature divergent patterns of variation between rivers and oxbow lakes (Table 1). In the process of community assembly, the influence of environmental filtering tends to increase with the variability of environmental factors, thus dominating further in those areas having high habitat heterogeneity (Jackson et al., 2001). An oxbow lake is a stable lentic system, which, together with rivers, forms a mosaic floodplain of unique environmental gradients (Ward, 1998; Wang et al., 2020). More importantly, low hydrological connectivity and runoff in the non-flood period could limit overall habitat availability and quality, perhaps acting as a “natural” environmental filter (Chase, 2007; Liu et al., 2016). Thus, environmental filtration (species ranking paradigm) was the dominant process driving community assembly in non-flood season. In the flood period, the predominant influence of environment filtering diminished, while spatial progresses now exerted a stronger influence upon phytoplankton community in the study area. This shift suggests that increased hydrological connectivity in the river-oxbow lake system enables high-frequency species migration events to occur between multiple local communities, thereby triggering mass effects and reducing dispersal limitation (Bortolini et al., 2017). In the mass effects paradigm, high dispersal rates allow species to colonize non-optimal habitats from optimal habitats, thus mitigating the biological control of environmental selection and ecological drift on communities. Moreover, the wide distribution range of phytoplankton indicates that dispersal limitation has overall only a small influence on spatial turnover in phytoplankton communities during the flood period (Brown and Swan, 2010; Niño-García et al., 2016).

The analysis of DDRs is a powerful method to detect species dispersal-driven dynamics indirectly from spatial community data (Brown and Swan, 2010). In the present study, we found that phytoplankton communities in the river (mainstream and tributaries) exhibited significant DDRs during the two hydrological periods (Figure 5), consistent with dispersal-driven dynamics. Furthermore, Mantel tests revealed that environmental factors significantly affected the metacommunity dynamics in rivers only during the non-flood season (Supplementary Table 3). This suggest that, in addition to dispersal-driven dynamics, species sorting paradigm was another important factor restricting the dynamics of river communities. Our results were agreeing with Brown and Swan (2010), who reported both dispersal-driven and environmental constraints on mainstream metacommunity structure. Conversely, high flow and hydrological connectively during the flood period promoted connections between river habitats, wakening the impact of environmental constraints on the community and enabling phytoplankton to be more easily and passively dispersed by water flow (Datry et al., 2016; Chen et al., 2019). Interestingly, there was a lack of significant DDRs for phytoplankton communities in oxbow lakes (Figure 5). The Mantel tests suggest the metacommunity in oxbow lakes would be predominantly structured following the species-sorting paradigm and governed by local environmental variables during the non-flood period (Clarke et al., 2008; Supplementary Table 3).

### 4.2. Seasonal and spatial variations of phytoplankton community in highland floodplain

Hydrological alterations can lead to temporal changes of organisms in the macrosystem of floodplains, including riverine, and lowland habitats that are connected and interacting at the watershed scale (Townsend, 2006; Butler et al., 2007; Yang et al., 2017). Therefore, alterations in hydrological conditions play an essential role in structuring phytoplankton communities in river-oxbow lake systems (Schemel et al., 2004). As expected, the NMDS results indicated the phytoplankton community composition was distinctly different between seasons and habitats. Several reasons could explain the significant seasonal changes in phytoplankton community. First, the environmental conditions varied drastically between the two periods as shown in Table 1. On the other hand, the phytoplankton communities may undergo the seasonal succession. Furthermore, we found that the habitat difference of phytoplankton lessened in the flood period. This may be because oxbow lakes are isolated from the river channel during the non-flood period, with prevailing characteristics such as low site-to-site connectivity and prolonged water retention time (Isabwe et al., 2018). Consequently, differences in local community composition among habitat types were relatively high. By contrast, during the flood period, oxbow lakes distributed in lowland plains were connected in tandem with the river channel by the flood pulse. This greater hydrological connectivity could accelerate phytoplankton dispersal and allow species to colonize non-optimal habitats from optimal habitats, thereby decreasing phytoplankton beta diversity in the river-oxbow lake system (Jiang et al., 2021). The above findings confirm that hydrological changes coupled to mass effects may drive phytoplankton community assembly in river-oxbow lake systems of highland floodplains (Read et al., 2015; Niño-García et al., 2016; Chaparro et al., 2018).

In addition, we found that phytoplankton density, biomass, and alpha diversity in the river and oxbow lake habitats all markedly decreased from the non-flood to the flood period (Table 2). To explain this phenomenon, we suggest several direct and indirect reasons for it. First, in the flood period, the mean nutrient concentrations decreased sharply, while turbidity increased in all three habitats of the White River Basin compared with those in the non-flood period (Supplementary Table 1). Nutrients play a key role in community dynamics by supporting phytoplankton growth (Muhid et al., 2013; Navas-Parejo et al., 2020), whereas high turbidity usually leads to reductions in euphotic depth and photosynthetically active radiation, which would directly limit phytoplankton growth (Cardoso et al., 2017; Wang et al., 2019). Second, increased stream flows and physical disturbances lead to phytoplankton washout and density-independent mortality of most phytoplankton species (Cook et al., 2010; Townsend and Douglas, 2017), Third, flooding has a profound influence on the macrophyte structure of the river-oxbow lake system in floodplains (Ibelings et al., 2007; Zhou et al., 2019). During the flood period, massive macrophyte vegetation would absorb a large portion of nutrients and produce allelopathic substances, which could suppress phytoplankton biomass accumulation to a low level by causing nutrient limitations (Gross et al., 2007).

### 4.3. Ecological implication for health management in highland floodplain ecosystems

There are thousands of oxbow lakes located in the Zoige Basin, together with rivers, providing an important role for biodiversity conservation and regional habitat heterogeneity maintenance in Tibetan Plateau floodplain. Our results showed that the highest richness values were found in oxbow lakes in both seasons (140 in the non-flood and 109 in the flood period). This confirms that oxbow lakes play a vital role for improving overall biodiversity in the highland floodplain system. Furthermore, we found that allochthonous genera (belonging to the phyla Cyanophyta and Chlorophyta) from oxbow lakes in the flood period potentially accounted for 16.3% of the total phytoplankton diversity in the mainstream, a much higher contribution than in the non-flood period (2.8%; Supplementary Figure 4). Generally, aquatic organisms may disperse from “source” sites (mainstream) to “sink” sites (oxbow lakes) along the horizontal direction of water flow. Dai et al. (2020) reported that allochthonous species from the Yangtze River potentially contributed to 25% of the phytoplankton diversity in Lake Taihu during periods of water diversion. Pan et al. (2022) found the coalescence of bacteria in oxbow lakes from mainstream and tributaries increased with increasing hydrological connectivity. However, our results are inconsistent with the previous results. This suggests that oxbow lakes, as water-receiving aquatic ecosystems, may become “source” sites under ephemeral conditions of high hydrological connectivity. Accordingly, the associated mass effects also are indispensable in shaping phytoplankton communities in complex aquatic networks.

However, in the past few decades, the floodplain oxbow lakes in the northeastern Tibetan Plateau have gradually shrunk and degraded into swampy grasslands due to the influence of global warming and human impact (Zhou et al., 2019; Wang et al., 2020). According to our field survey, some shallow oxbow lakes have an average water depth of only 10–15 cm, which could evaporate completely in as little as a week. This also foreshadows the loss of aquatic organisms inhabiting in this habitat and the consequent instability of the ecosystem. Therefore, the relevant management strategy should be required to maintain the oxbow lakes as important habitats for biota in highland floodplains.

## 5. Conclusion

The present study disentangled the mechanisms driving the spatiotemporal patterns of phytoplankton communities in a river-oxbow lake system of the Tibetan Plateau floodplain across hydrological periods. The contribution of environmental filtering and spatial processes for phytoplankton community assembly was revealed. We observed distinct phytoplankton community composition between rivers and oxbow lakes in the contrasting hydrological periods. During the flood period, the flood pulse considerably reduced phytoplankton density, biomass, and alpha diversity. The improved hydrological connectivity between rivers and oxbow lakes lessened environmental heterogeneity among different habitats, thus promoting species dispersal and consequently reducing beta diversity. In both hydrological periods, phytoplankton communities in rivers displayed distance-decay patterns, whereas the distance–decay relationships were stronger during the non-flood than flood period. Environmental filtering chiefly governed phytoplankton community structure in the non-flood period, but the most influential environmental factors varied between hydrological periods. In addition, the role of spatial processes was stronger than environmental filtering for phytoplankton in the flood period. In addition to stochastic factors, future ecological studies should consider other explanatory factors (i.e., a wider range of environmental gradients and interspecific interactions).

## Data availability statement

The original contributions presented in this study are included in this article/Supplementary material, further inquiries can be directed to the corresponding author.

## Author contributions

ZH: formal analysis, writing—original draft, and software. BP: conceptualization, supervision, and writing—review and editing. JS: writing—review and editing. XinyL: methodology and formal analysis. XingL: data curation and software. YH: data curation and validation. All authors contributed to the article and approved the submitted version.
