# Supplementary material for: Seasonal variation of phytoplankton community assembly processes in Tibetan Plateau floodplain
Source: Front Microbiol. 2023 Feb 20;14:1122838. doi: 10.3389/fmicb.2023.1122838 (PMC9986264; doi:10.3389/fmicb.2023.1122838)
Supplement: Supplementary file 1 [file Table_1.DOCX]

**Supplementary material for**

**Seasonal variation of phytoplankton community assembly processes in Tibetan** **plateau floodplain**

**Zhenyu Huang; Baozhu Pan*; Janne Soininen; Xinyuan Liu; Yiming Hou; Xin Liu;**

**Table of Content**

**Table S1** Environmental variables in the mainstream, tributaries, and oxbow lakes of the White River Basin during two contrasting hydrological periods.

**Table S2** ANOSIM results showing phytoplankton community differences in different habitats of the White River Basin during the two contrasting hydrological periods.

**Table S3** Mantel tests showing Pearson’s correlations between phytoplankton community and environmental and geographic distance.

**Fig. S1.** Daily runoff in the White River Basin during the non-flood and flood periods.

**Fig. S2.** PERMDISP analyses based on Euclidean distance of environmental data from different seasons, showing the mean distance to group [centroid](https://www.sciencedirect.com/topics/earth-and-planetary-sciences/centroid) in each season.

**Fig. S3.** Relative abundances of phytoplankton densities in three different habitats during the (a) non-flood period and (b) flood period of the White River Basin.

**Fig. S4.** Venn diagrams showing the numbers of unique and shared phytoplankton genera among the mainstream, tributaries, and oxbow lakes of the White River Basin during the two contrasting hydrological periods.

**Table S1**

Environmental variables (mean ± standard deviation) in the mainstream, tributaries, and oxbow lakes of the White River Basin during two contrasting hydrological periods.

| Variable | Mainstream | | Tributaries | | Oxbow lakes | |
| --- | --- | --- | --- | --- | --- | --- |
|  | Non-flood period | Flood period | Non-flood period | Flood period | Non-flood period | Flood period |
| WD (cm) | 21.39±1.34 | 34.22±4.86 | 18.49±1.64 | 27.93±2.90 | 23.83±2.90 | 28.48±2.29 |
| V (m/s) | 0.44±0.05 | 0.58±0.13 | 0.58±0.08 | 0.44±0.10 | - | - |
| pH | 8.09±0.36 | 7.56±0.03 | 7.84±0.09 | 7.60±0.04 | 7.93±0.19 | 7.39±0.08 |
| WT (°C) | 13.55±0.72 | 13.52±0.80 | 12.57±0.78 | 13.87±0.57 | 14.48±0.98 | 15.40±0.42 |
| Cond (μs/cm) | 88.28±5.77 | 57.31±2.99 | 104.78±6.31 | 87.48±14.19 | 98.13±6.87 | 64.43±4.69 |
| DO (mg/L) | 6.52±0.13 | 5.49±0.24 | 6.60±0.15 | 5.33±0.21 | 5.71±0.48 | 4.58±0.24 |
| Tur (NTU) | 173.34±54.91 | 564.27±133.46 | 110.42±29.23 | 339.81±52.84 | 58.02±14.41 | 175.45±30.71 |
| TN (mg/L) | 1.07±0.10 | 0.33±0.04 | 0.82±0.05 | 0.55±0.07 | 0.98±0.06 | 0.43±0.06 |
| NH_4_^+^-N (mg/L) | 0.87±0.09 | 0.2±0.04 | 0.68±0.06 | 0.33±0.06 | 0.68±0.09 | 0.16±0.03 |
| NO_3-_^—^N (mg/L) | 0.03±0.01 | 0.03±0.01 | 0.03±0.01 | 0.03±0.01 | 0.04±0.01 | 0.04±0.01 |
| TP (mg/L) | 0.08±0.01 | 0.03±0.01 | 0.06±0.01 | 0.03±0.01 | 0.08±0.01 | 0.05±0.01 |

WD, water depth; V, flow velocity, Cond, electrical conductivity; DO, dissolved oxygen; Tur, turbidity; TN, total nitrogen; NH_4_-N, ammonia nitrogen; NO_3_-N, nitrate nitrogen; TP, total phosphorus.

**Table S2**

ANOSIM results showing phytoplankton community differences in different habitats of the White River Basin during the two contrasting hydrological periods.

|  | Habitat group | Distance | *r* | *P* |
| --- | --- | --- | --- | --- |
| Non-flood  period | All | Bray-Curtis | 0.389 | 0.001 |
|  | Mainstream vs. Tributaries |  | 0.164 | 0.023 |
|  | Mainstream vs. Oxbow lakes |  | 0.434 | 0.001 |
|  | Tributaries vs. Oxbow lakes |  | 0.423 | 0.001 |
| Flood period | All | Bray-Curtis | 0.338 | 0.001 |
|  | Mainstream vs. Tributaries |  | 0.223 | 0.006 |
|  | Mainstream vs. Oxbow lakes |  | 0.350 | 0.005 |
|  | Tributaries vs. Oxbow lakes |  | 0.389 | 0.003 |

The *r* values closer to 0 represent little or no differences in community composition, while values close to 1 represent large differences.

**Table S3**

Mantel tests showing Pearson’s correlations between phytoplankton community dissimilarity and environmental and geographic distance.

| Period | Factors | Mainstream | tributaries | Oxbow lakes |
| --- | --- | --- | --- | --- |
| Non-flood period | Geographic distance | 0.386* | 0.293* | 0.108 |
|  | Environmental distance | 0.266* | 0.351* | 0.215* |
| Flood period | Geographic distance | 0.269* | 0.171 | 0.068 |
|  | Environmental distance | 0.135 | 0.059 | 0.029 |

Note: The symbol * represents the p-value < 0.05.


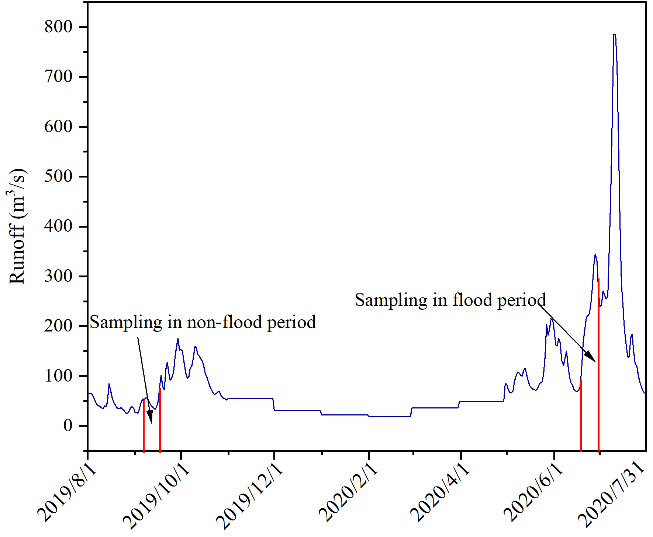


**Fig. S1.** Daily runoff in the White River Basin during the non-flood and flood periods.


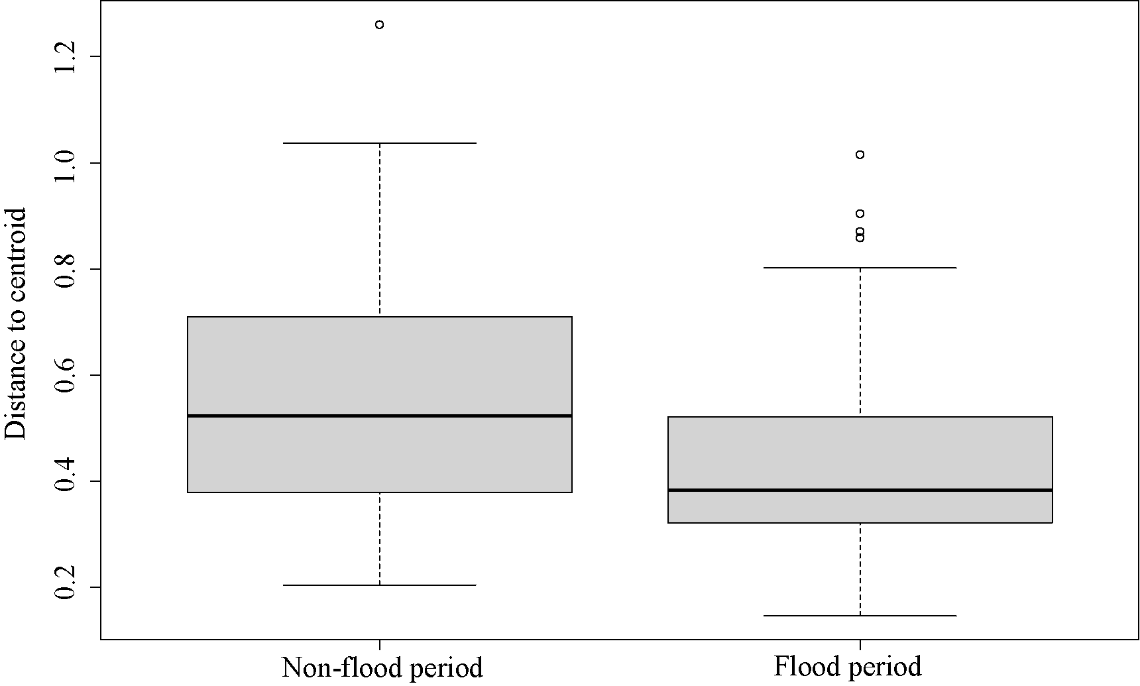


**Fig. S2.** PERMDISP analyses based on Euclidean distance of environmental data from different seasons, showing the mean distance to group [centroid](https://www.sciencedirect.com/topics/earth-and-planetary-sciences/centroid) in each season.


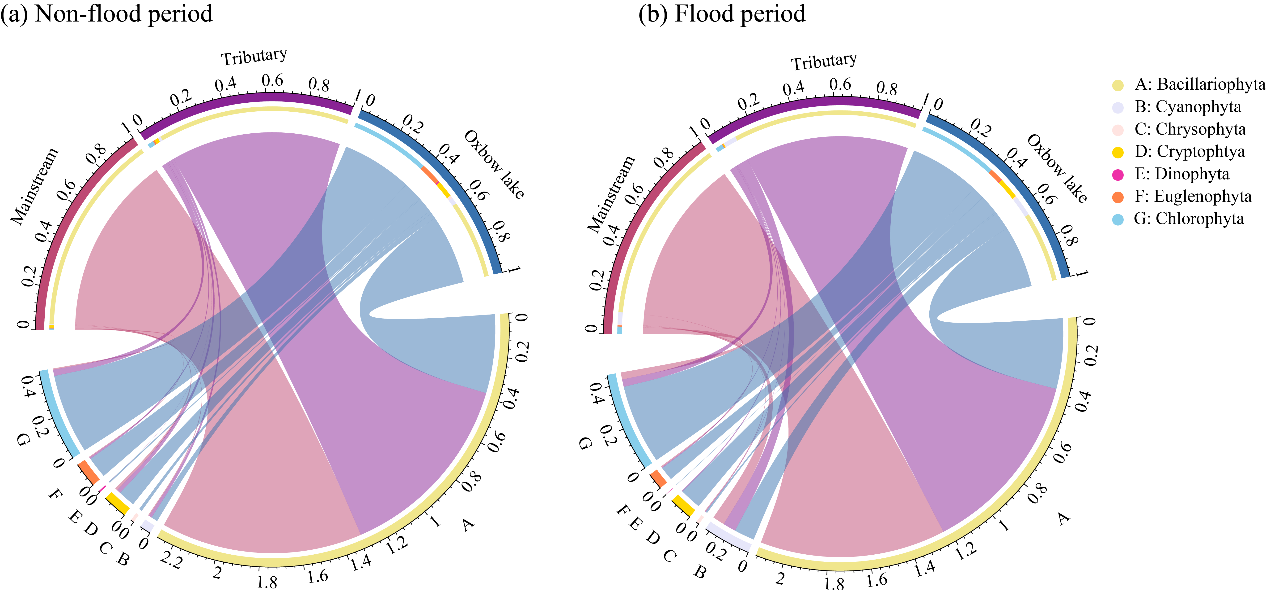


**Fig. S3.** Relative abundances of phytoplankton densities in three different habitats during the (a) non-flood period and (b) flood period of the White River Basin.


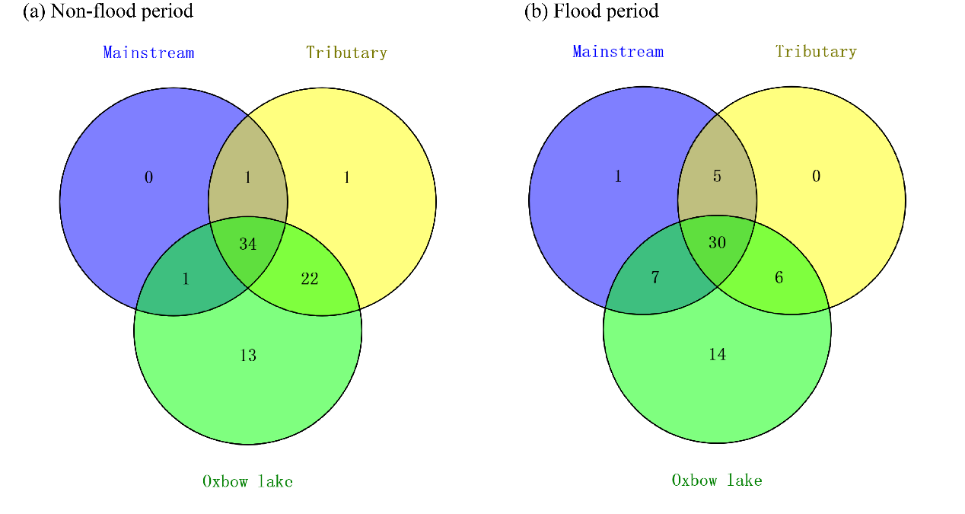


**Fig. S4.** Venn diagrams showing the numbers of unique and shared phytoplankton genera among the mainstream, tributaries, and oxbow lakes of the White River Basin during the two contrasting hydrological periods.
